# Clinical factors affecting prostate-specific antigen levels in prostate cancer patients undergoing radical prostatectomy: a retrospective study

**DOI:** 10.2144/fsoa-2020-0154

**Published:** 2021-01-12

**Authors:** Giovanni Tarantino, Felice Crocetto, Concetta Di Vito, Raffaele Martino, Savio Domenico Pandolfo, Massimiliano Creta, Achille Aveta, Carlo Buonerba, Ciro Imbimbo

**Affiliations:** 1 Department of Clinical Medicine & Surgery, Federico II University Medical School of Naples, 80131 Naples, Italy; 2 Department of Neurosciences, Reproductive & Odontostomatological Sciences, Urology & Andrology Unit, Federico II University of Naples, 80131 Naples, Italy; 3 Department of Oncology & Hematology, Regional Reference Center for Rare Tumors, AOU Federico II of Naples, 80131 Naples, Italy

**Keywords:** BMI, COPD, Gleason score, heavy drinking, smoking habit, total PSA

## Abstract

**Background:**

Since prostate-specific antigen (PSA) levels can be influenced by some routinely available clinical factors, a retrospective study was conducted to explore the influence of obesity, smoking habit, heavy drinking and chronic obstructive pulmonary disease on PSA levels in men with histologically confirmed prostate cancer.

**Patients & methods:**

We reviewed the medical records of 833 prostate cancer patients undergoing radical prostatectomy.

**Results:**

Serum PSA levels at the time of surgery were not associated with either BMI or history of chronic obstructive pulmonary disease or heavy drinking. Conversely, PSA levels were associated with smoking status.

**Conclusion:**

Among the clinical factors explored in this homogeneous population, only tobacco use was associated with PSA levels, which should be considered when using PSA-based screening in male smokers.

## Lay abstract

Smokers with prostate cancer tend to show higher PSA levels at the time of radical prostatectomy. As higher PSA levels are associated with a worse prognosis, smoking habit may have a prognostic value in prostate cancer. Further studies are required to explore the underlying biology of this finding.

Prostate cancer is one of the most frequently diagnosed cancers in men, and a major cause of cancer-related death. Localized disease can be effectively treated with surgery and/or radiotherapy [1], while advanced disease must be treated with chemotherapy and hormone therapy [2,3]. The standard screening strategy for prostate cancer includes digital rectal examination (DRE) and assessment of prostate-specific antigen (PSA) levels, which correlate with Gleason score [4,5]. Physicians must be well aware of the limitations of PSA-based screening, which suffers from a high rate of false-positive results and false-negative results as well as from a high risk of performing unnecessary biopsies and even treatment. Indeed, consensus is lacking on whom to screen, when to screen and what to do if cancer is discovered. PSA-based screening misses approximately 18–25% of prostate cancers and provides false-positive results in approximately 60% of cases [6]. In this scenario, research on factors affecting PSA levels is of primary importance. Older age and lower BMI are associated with higher PSA levels [7–9] and lower levels of PSA in obese and overweight men could make PSA-based screening even less reliable in overweight men [10]. This may be due to lower testosterone levels or greater plasma volume in obese men [11]. Besides obesity, hypertriglyceridemia, hyperglycemia and low levels of high-density lipoprotein cholesterol are associated with decreased PSA levels [12]. Smoking may also play a role in influencing PSA levels. PSA levels are significantly higher in smokers than in nonsmokers in prostate cancer patients [13], which may be mediated by smoking-induced endocrine dysfunctions. Furthermore, chronic obstructive pulmonary disease (COPD), the third-leading cause of death, has been associated with testosterone deficiency in men and decreased PSA levels [14]. Finally, alcohol consumption may also influence PSA levels [15].

Even though several biological mechanisms potentially link obesity, smoking, COPD and alcohol consumption to prostate cancer, their effect on serum PSA levels is yet to be fully elucidated. As PSA still represents the main screening tool for prostate cancer, factors influencing PSA levels should be investigated and considered. In this retrospective study, we aimed to explore the relationship between selected commonly available clinical variables and PSA levels in surgical prostate cancer series.

## Patients & methods

### Study design

In this retrospective study, we reviewed the medical records of prostate cancer patients who were diagnosed and treated with prostatectomy according to standard clinical practice. All extracted data were anonymized for analysis. Ethical approval or informed written consent was unnecessary for this retrospective study.

Patients who underwent surgery for histologically confirmed prostate adenocarcinoma were included. Patients presenting the following systemic diseases were excluded (American Society of Anaesthesiologists physical status 3): poorly treated hypertension or diabetes mellitus, morbid obesity, chronic renal failure, severe bronchospastic disease with intermittent exacerbation, stable angina and implanted pacemaker, according to Knuf *et al.* [16].

Diagnosis of prostate cancer relied on transrectal ultrasound (TRUS) followed by biopsy. Assessment of total serum PSA levels was performed before surgery.

### Transrectal ultrasound

Inspection of the gland focused on identifying asymmetry, areas of increased vascularity, hypoechogenicity and the presence of focal bulges, irregularity, or breaches of the capsule. These features were reckoned as associated with the presence of cancer but were not sufficiently reliable to make a diagnosis without obtaining a biopsy. The gland volume was calculated using an ellipsoid formula by measuring the maximum anteroposterior, craniocaudal and transverse distances and multiplying the product of these by π/6 [17].

### Biopsy

The biopsy protocol included 10–12 needle core biopsies (18 Fr gauge) for systematic mapping of the prostate, including any palpable or radiological target lesions. Patients were histologically confirmed by biopsy or transurethral resection of the prostate. Prostate volume in cm^3^ was measured by planimetry during the biopsy procedure.

Indications for prostate biopsy were a PSA >4.0 ng/ml, an abnormal DRE/TRUS or both [18].

### Grading of prostate cancer adenocarcinoma

The standard grading of prostate cancer was performed according to the Gleason grading system (score from 1 [least aggressive] to 5 [most aggressive]) on the largest available histological specimen, either a biopsy or after radical prostatectomy, a whole prostate. The two most common Gleason patterns (primary and secondary) are added to give a total score ranging from 2 (1 + 1) to 10 (5 + 5).

### Lymph node staging

We adhered to the conventional approach to lymph node (LN) staging in prostate cancer patients, including the use of contrast-enhanced computed tomography (CT) and MRI. For both modalities, the definition of suspicious LNs was based prevalently on size thresholds for enlarged LNs. The most frequently used threshold was 10 mm in short-axis diameter [19].

### Staging for bone metastases

Patients were thought to have formally made the transition to having bone metastatic disease when bone scintigraphy gave the presence of bone metastasis(es). Whole-body bone scintigraphy was performed using Tc-99-methylenediphosphonate and reviewed by a certified nuclear medicine physician with extensive experience. The skeletal metastasis on bone scintigraphy was defined as either solitary or multiple asymmetric areas of increased tracer uptake presence, excluding tracer accumulations related to previous trauma and degenerative bone disease [20]. Patients’ equivocal bone scan findings also underwent CT or MRI to confirm the bone scintigraphy findings.

### Anthropometric measures

BMI was categorized as follows: <25 kg/m^2^ (normal weight); 25–29.9 kg/m^2^ (overweight); and ≥30 kg/m^2^ (obese), according to WHO (Geneva, Switzerland) classification.

### Diagnosis of diabetes mellitus

Type 2 diabetes mellitus was diagnosed if a random blood sugar level was ≥200 mg/dl, or a fasting plasma glucose level was ≥126 mg/dl (7.0 mmol/l), or a 2-h plasma glucose level was ≥200 mg/dl (11.1 mmol/l) during a 75-g oral glucose tolerance test or hemoglobin A1C was 6.5%.

Type 1 diabetes mellitus was diagnosed on the presence of serum autoantibodies, in other words, islet cell autoantibodies and ketones in patients’ urine.

### Diagnosis of hypertension

Systolic and diastolic blood pressures were ≥130 and ≥85 mmHg, respectively; both measurements were taken in a seated position after resting for at least 5 min.

### Smoking habit

Patients were classified as no smokers/former smokers and active smokers. Active smokers were classified as light smokers (<five cigarettes/day); moderate smokers (5–15 cigarettes/day) and heavy smokers (>15 cigarettes/day).

COPD was diagnosed on the basis of personal and medical history, chest radiography and pulmonary function tests.

### Drinking habit

The definition for alcohol abuse according to US Centers for Disease Control and Prevention Dietary Guidelines was followed.

### Laboratory data

Total PSA levels were assessed by a chemiluminescence immunoassay according to the manufacturer’s indications.

### Statistics

Data were described as mean plus standard deviation if showed a normal distribution, while not normally distributed continuous variables or ordinal variable were described as median plus 25–75 interquartile range.

Frequency tables were used to assess relationships between Gleason patterns, assessing the Pearson chi square and the p-value.

The difference between medians was, as analyzed by the two-sample Wilcoxon rank-sum (Mann–Whitney) test, as measure of association, we chose to study predictions that were carried out by various types of regression techniques. As univariate analysis the linear regression analysis was employed. Multivariate analysis was performed to account for multiple variables including age.

To provide a measure of how well observed outcomes are replicated by the model, based on the proportion of total variation of outcomes explained by the model, the R-squared was computed.

In suspicion of heteroscedasticity and having detected the presence of few outliers, we analyzed the correlation by the ‘robust’ regression, using Least Absolute Deviations regression.

An ordered probit model was arranged to estimate predictions between an ordinal dependent variable, expressed as grades (0–3) and a set of independent variables. The output showed the coefficients, their standard errors, the z-statistic (also called a Wald z-statistic) and the associated p-values.

Dealing with a binary dependent variable the prediction tool carried out was the logistic regression by which the odds ratio with related 95% CI was evaluated.

The Kendall score was used to compare a set of pair observations, analyzing Tau-a and Tau-b. DRE was categorized as positive/negative. When the TRUS suggested prostate cancer, the case was classified as abnormally present and categorized as yes/not. Stata16.0 was the program on which we run statistics.

## Results

Main characteristics of the studied population are shown in Table 1. Noteworthy, the most frequent comorbidity was hypertension, while median Gleason score was 7.

**Table 1.** Characteristics of the study cohort.

Number of observations (pts)	833
Age (years), median (25–75 IQR)	66 (61–70)
T2DM (n of pts)	79
T1DM (n of pts)	2
Hypertension (n of pts)	421
COPD (n of pts)	53
Hyperuricemia (n of pts)	9
Hypertriglyceridemia (n of pts)	46
Hypercholesterolemia (n of pts)	134
BMI total population, median (IQR)	26 (25–28.7)
BMI ≥25 (n of pts)	497^†^
BMI ≥30 (n of pts)	105
Gleason score median (25–75 IQR)	7 (6–7)
Serum PSA of the whole population	7.4 (5.5–10.4) ng/ml
Serum PSA in overweight pts	7.25 (5.3–10) ng/ml
Serum PSA in obese pts	8 (5.7–11.65) ng/ml
Metastatic cancer, n of pts with LNs^†^	1/288
Metastatic cancer, n of pts with BM^†^	38/588
Smoking habit (yes/no), n of pts^†^	530/145
Smoking characteristics, categorized as 1/2/3 (n of pts)^†^	19/45/43
Heavy drinking habit yes/not (n of pts)^†^	27/615

† Some patients of the cohort were not evaluable for this characteristic.

0: No smokers; 1: Light smokers (<five cigarettes/day); 2: Moderate smokers (5–15 cigarettes/day); 3: Heavy smokers >15 cigarettes/day.

BM: Bone metastasis; COPD: Chronic obstructive pulmonary disease; IQR: Interquartile range; LN: Lymph node; PSA: Prostate-specific antigen; pt: Patient; T1/2DM: Type 1/2 diabetes mellitus.

Median of PSA serum levels were significantly higher in smokers compared with nonsmokers, that is 8 (5.8–11.7) versus 7 (5.4–9.7) (p = 0.004, two-sample Wilcoxon rank-sum, Mann–Whitney test). Interestingly, in this population of patients with prostate cancer, logistic regression showed that smoking habit was significantly associated with PSA levels (odds ratio = 1.04; standard error = 0.012; z = 3.66; 95% CI: 1.02–107). This association was independent on the number of cigarettes/day.

**Figure 1.**
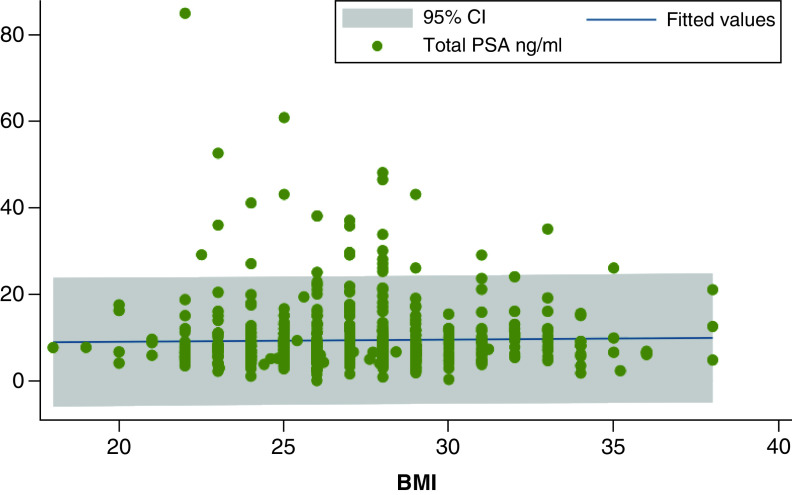
Distribution of total prostate-specific antigen levels according to BMI. The flat regression line indicates no statistical significance. PSA: Prostate-specific antigen.

There was no association between PSA values with BMI in the whole population (n = 648, p = 0.61, Figure 1), in the combined overweight and obese group (n = 495, p = 0.40) and in the obese subgroup (n = 105, p = 0.42). No association was found between PSA levels and COPD or with heavy drinking habit. As expected, levels of total PSA consistently were significantly associated with both positive DRE and with Gleason score, which was not associated with positive history for diabetes mellitus, hypertension, hypertriglyceridemia, hypercholesterolemia and hyperuricemia (data not shown).

## Discussion

The main finding of this study is that smoking habit was associated with total PSA levels, independently on the number of daily smoked cigarettes. Conversely, total PSA levels were not associated with history of COPD or alcohol abuse. Total PSA levels were associated with Gleason score, which was not associated with history of diabetes mellitus, hypertension, hypertriglyceridemia and hypercholesterolemia.

In this study cohort, we could not confirm that total PSA levels were associated with being overweight or obese, as reported by others [1,21,22].

As far as smoking habit is concerned, results are in line with published literature, although we could not confirm that smoking is associated with an increased risk of cancer-specific death [23–25]. The association of smoking and prostate cancer risk may have either a hormonal or genetic basis. Cigarette smoking affects various hormone levels and male smokers usually have higher levels of circulating sex hormones, which may increase prostate cancer risk or contribute to cancer progression [26–28]. Also, functional polymorphisms in genes involved in polycyclic aromatic hydrocarbons metabolism may affect cancer onset and progression [29]. Nevertheless, some authors did not find a direct correlation between smoking status and prostate cancer [30,31].

An increased risk of prostate cancer was found among COPD patients suffering from related complications such as acute respiratory failure, cardiopulmonary arrest, pneumonia and acute exacerbation [32].

Similarly, patients with COPD, particularly those using short-acting inhaled pharmacotherapy, have been shown to be at a higher risk of prostate cancer [33]. In our study cohort, COPD was not associated with total PSA levels. Although older patients with COPD had significantly lower total PSA compared with the control group, there was no significant difference in terms of PSA levels after adjusting for COPD severity. In keeping with previous findings [30], heavy versus light smokers did not appear to have different PSA levels.

Interestingly alcohol abuse did not affect total PSA values unlike previous findings [34].

As for other comorbidities such as diabetes mellitus, hypertension, dyslipidemia and hyperuricemia, our data concerning the severity of prostate cancer are in agreement with other observations, showing that the only significant predictor of death from prostate cancer was clinical Gleason score, while age and Charlson comorbidity score were significant independent predictors of death from other causes [35].

The strengths of this study consist in the large population assessed and its homogeneity in terms of cancer diagnosis and treatment. Its weaknesses are mainly due to its retrospective design and the lack of relevant data including prostate health index [36], testosterone levels [37] as well as physical activity [38,39]. From a general point of view, as in any observational study, the role of other potentially confounding factors not taken into account must be acknowledged.

## Conclusion

In this homogeneous population, tobacco consumption was the only clinical factor that was associated with PSA levels. Such an association should be considered when using PSA-based screening for prostate cancer as well as when assessing the risk group in patients undergoing prostatectomy.

## Future perspective

Smoking exerts a wide spectrum of negative effects in humans that may go beyond its well-known carcinogenic effects. Further studies are required to explore the prognostic value of smoking status in prostate cancer as well as smoking influence on accuracy of PSA-based screening for prostate cancer. As an example, the effect of PSA-based screening should be reconsidered in the smoking subgroup of the European Randomized Study of Screening for Prostate Cancer (ERSPC).

**Summary points**

- Prostate-specific antigen (PSA) is a protein synthesized by normal and cancer cells. PSA is mostly detected in semen, but a small amount is also found in blood and is measured in ng/ml.
- The likelihood of having prostate cancer increases as the PSA levels increase, but there is no set cut-off point that can assure if someone does have prostate cancer.
- Generally a PSA cut-off point of ≥4 ng/ml is used when deciding whether patient might need further testing, while others might recommend it.
- One reason for which it is difficult to use a set cut-off point with the PSA test when looking for prostate cancer is that a number of factors other than cancer can also affect PSA levels.
- In this study, the relationship of PSA levels with data regarding medical history was investigated to identify commonly available factors capable to influence PSA levels in prostate cancer patients.
- Among the clinical factors explored in this homogeneous population, only tobacco use was associated with PSA levels, which should be considered when using PSA-based screening in male smokers.
